# Chicago Dental Society

**Published:** 1894-05

**Authors:** H. A. Costner


### Chicago Dental Society.

At the annual meeting of the Chicago Dental Society, held in Kindergarten Hall, April 3, 1894, the following officers were duly elected for the ensuing year :

J. H. Woolley, President; C. E. Bentley, 1st. Vice-President; D. M. Gallie, 2d. Vice-President; A. H. Peck, Recording Secretary; H. A. Costner, Corresponding Secretary; E. D. Swain, Treasurer; J. J. Whaley, Librarian; J. M. Crouse, Director.

Yours truly,
H. A. Costner, Cor. Sec.

